# Dynamic Hippocampal–Striatal Information Flow Accompanies Behavioral Strategy Transitions During Sequential Learning in Pigeons: A Preliminary Study

**DOI:** 10.3390/ani16172755

**Published:** 2026-09-02

**Authors:** Lifang Yang, Ying Ma, Zhihui Li, Mengmeng Li

**Affiliations:** 1 School of Electrical and Information Engineering, Zhengzhou University, Zhengzhou 450001, China; lifang_yang1014@zzu.edu.cn (L.Y.); my20011011@163.com (Y.M.); lizhrain@zzu.edu.cn (Z.L.); 2 Henan Key Laboratory of Brain Science and Brain-Computer Interface Technology, Zhengzhou 450001, China; 3 The Affiliated Encephalopathy Hospital of Zhengzhou University, Zhumadian 463000, China

**Keywords:** sequential decision-making, behavioral strategy transition, model-based and model-free learning, hippocampal–striatal communication, directed information flow, pigeons

## Abstract

Sequential decision-making requires animals to flexibly balance model-based (MB) and model-free (MF) strategies to adapt to changing environments. The hippocampus (Hp) and striatum (ST) are two important components of the broader neural networks supporting these processes; however, how their dynamic interactions reorganize during learning-dependent strategy transitions remains poorly understood. Here, we trained pigeons on a two-step sequential decision-making task while simultaneously recording local field potentials (LFPs) from the Hp and ST. A dynamic reinforcement learning framework combined with a sliding-window approach was used to characterize temporal changes in behavioral strategies, and phase transfer entropy (PTE) was applied to estimate directed information flow between the Hp and ST across theta, beta, and broad gamma (30–80 Hz) frequency bands. Behavioral modeling revealed a gradual transition from early MB-like, task-structure-sensitive control toward later MF-like value-guided behavior as learning progressed. PTE analysis demonstrated a consistent Hp-to-ST directional bias across all analyzed frequency bands during task acquisition. Notably, gamma-band Hp-to-ST information flow exhibited a consistent decline over training, whereas theta- and beta-band interactions showed less consistent changes across individuals. Additional analyses showed that relative MB model evidence and gamma-band Hp-to-ST information flow covaried across learning, but this association was no longer significant after controlling for learning progression, indicating parallel rather than independently coupled changes. These preliminary findings indicate that hippocampal–striatal communication undergoes frequency-specific reorganization during sequential learning. The reduction in gamma-band Hp-to-ST information flow accompanies, rather than independently predicts, the behavioral strategy transition, suggesting learning-related modulation of interregional coordination as task demands change.

## Simple Summary

Animals adapt their decisions by combining current information with previous experience. During learning, they may shift from flexible strategies based on environmental structure toward more stable choices guided by learned values. In this study, pigeons performed a sequential decision-making task while neural activity was recorded from the hippocampus and striatum. Behavioral modeling showed a gradual shift toward value-guided choice, accompanied by changes in communication between these brain regions. In particular, gamma-band information flow from the hippocampus to the striatum decreased as task performance became more stable. These preliminary findings suggest that learning is accompanied by frequency-specific changes in brain communication and provide new insights into the neural basis of adaptive decision-making in birds.

## 1. Introduction

In complex and uncertain environments—specifically those characterized by probabilistic reward contingencies and dynamic state-transition rules—animals must adapt their behavior by integrating current cues with previous experience. Sequential decision-making therefore requires both the evaluation of immediate outcomes and the learning of transition relationships across decision stages. With repeated experience, behavioral control may become increasingly dependent on previously learned action values and habit-related striatal processes [1]. Reinforcement learning (RL) provides a formal framework for describing these changes, most commonly through Model-based (MB) and Model-free (MF) learning [2]. MB control uses an internal model of state transitions and outcomes to support flexible planning, whereas MF control relies on accumulated value estimates to guide efficient choices. Balleine and O’Doherty identified dissociable corticostriatal systems for goal-directed and habitual actions, involving the dorsomedial and dorsolateral striatum, respectively [3]. Piray and Daw further proposed a linear RL framework that links flexible planning with efficient value-based computation [4], while McNamee et al. showed that sequence representations in the entorhinal–hippocampal system can be flexibly adjusted according to task demands [5]. Hippocampal experience replay has also been proposed as a mechanism supporting planning and future-oriented decision-making [6].

The investigation of avian cognitive abilities rests upon a rich historiographical tradition, having experienced a period of particular flourishing owing to the pioneering work of B. F. Skinner and subsequent generations of scholars [7,8]. For decades, laboratory pigeon research served as the bedrock for studies of ‘learning and memory,’ frequently acting as a comparative counterpart to analogous procedures conducted in white rats and mice. Acknowledging this historical context is highly relevant, as it underscores the foundational role of avian research in elucidating the core mechanisms of animal and human cognition. Today, by applying modern analytical technologies—such as dynamic reinforcement learning models inspired by artificial intelligence (AI) and multichannel electrophysiology [9]—we can revisit and reinterpret these classic operant conditioning and sequential learning paradigms. This modern approach allows us to uncover the intricate neural network dynamics that underlie the behavioral phenomena first described by early cognitive pioneers.

At the neural level, rather than acting as isolated command centers, the hippocampus (Hp) and striatum (ST) function as highly integrated nodes within a broader distributed network governing learning and decision-making [10]. In contemporary avian neuroscience, these regions are understood to provide complementary computational roles. The Hp contributes to episodic memory, state representation, and the construction of cognitive maps of environmental structures [11,12,13], whereas the ST is involved in reward prediction, value updating, action selection, and reinforcement-based behavioral regulation [14,15,16]. Consistent with this role, recent studies in pigeons have shown that striatal network coordination tracks option value during probabilistic choice, indicating that avian striatal circuits contribute to value-based behavioral regulation [17]. Similarly, the pigeon hippocampus has been shown to contribute to model-based valuation and the representation of temporal contextual states, suggesting that avian hippocampal circuits support flexible decisions based on environmental structure [18]. These two brain regions are functionally distinct. This distinctiveness provides a dual-system foundation, allowing the brain to simultaneously process complex cognitive representations and efficient stimulus-response mappings [19]. Recordings during spatial decision-making have demonstrated distinct information processing across the Hp and ST subregions [20], and vicarious trial and error (VTE) has been associated with prospective evaluation during deliberation [21]. DeCoteau et al. found that striatal activity became increasingly coordinated with hippocampal theta rhythms during procedural learning [22]. Daw et al. further showed that in a two-stage task, model-based information influences not only behavioral choices but also striatal prediction-error signals [23]. In reinforcement learning, a prediction-error signal represents the mismatch between an expected reward and the actual reward received. The finding that model-based knowledge modulates these signals indicates that higher-level planning can directly shape the brain’s fundamental reward-learning mechanisms, while subsequent evidence has emphasized complementary Hp–ST contributions to flexible navigation and decision-making [24].

Recent studies further indicate that behavioral strategies can coexist and change dynamically over time. Le et al. identified mixtures of model-free and inference-based strategies during reversal learning [25], whereas Venditto et al. described time-dependent changes in strategy use during reward learning [26]. At the circuit level, interregional communication is partly organized through frequency-specific oscillatory coordination. The communication-through-coherence framework proposes that phase alignment regulates the effectiveness of information exchange between neural populations [27]. Nevertheless, it remains unclear how directed Hp–ST interactions reorganize as learning progresses, whether these changes are frequency-specific, and how they relate to the transition from flexible structure-based control to more stable value-guided behavior.

To address these questions, we simultaneously recorded local field potentials (LFPs), which reflect the collective electrical activity of local neuronal populations, from the Hp and ST of pigeons performing a two-step sequential decision-making task that required integration of state-transition information and reward outcomes. We tested whether Hp–ST communication showed a consistent directional bias and whether its strength changed as behavioral control shifted from early MB-like, task-structure-sensitive strategies toward later MF-like, value-guided behavior. We specifically hypothesized that Hp→ST information flow would predominate during learning and that gamma-band interactions would decrease as task performance became more stable. We further examined whether changes in gamma-band information flow covaried with the relative evidence for MB-like and MF-like behavioral strategies. Analyses of theta- and beta-band interactions were treated as exploratory to characterize the broader frequency-specific organization of Hp–ST communication across learning.

## 2. Materials and Methods

### 2.1. Experimental Animals and Surgery

Four adult homing pigeons (*Columba livia*; 450–550 g; approximately 1–2 years old, sex undetermined) were used in this study. The birds were obtained from Gongchuang Pigeon Farm, Zhengzhou, Henan Province, China, and were derived from a standard domestic lineage. None of the pigeons had prior experience with the present two-step sequential decision-making task before task-specific training. The animals were housed in an environmentally controlled facility (3 m × 3 m × 2 m) with natural light and adequate ventilation. During behavioral training and recording periods, food intake was restricted to maintain motivation for task performance, while water was available ad libitum. All experimental procedures were approved by the Life Sciences Ethics Review Committee of Zhengzhou University (No. SZZUIRB2022-44) and were performed in accordance with institutional guidelines for the care and use of experimental animals.

For chronic electrophysiological recording, pigeons were anesthetized with 2% sodium pentobarbital (0.2 mL/100 g body weight) and positioned in a custom stereotaxic apparatus [28]. The anterior fixation point (beak bar) was adjusted 45° below the horizontal plane to match the stereotaxic coordinate system of the pigeon brain atlas. Two 16-channel tungsten microwire electrode arrays (32 channels in total; wire diameter: 35 μm; inter-electrode spacing: 300 μm; Kedou, Suzhou, China) were chronically implanted into the Hp and ST. According to the electrode-array configuration, channels 1–16 corresponded to the Hp and channels 17–32 to the ST. The implantation coordinates were determined according to the pigeon brain atlas of Karten and Hodos. The coordinates were as follows: Hp: anteroposterior (AP) +5.5 mm, mediolateral (ML) ±1.0 mm, dorsoventral (DV) 1.0 mm; ST: AP +10.5 mm, ML ±1.0 mm, DV 7.5 mm.

**Figure 1:**
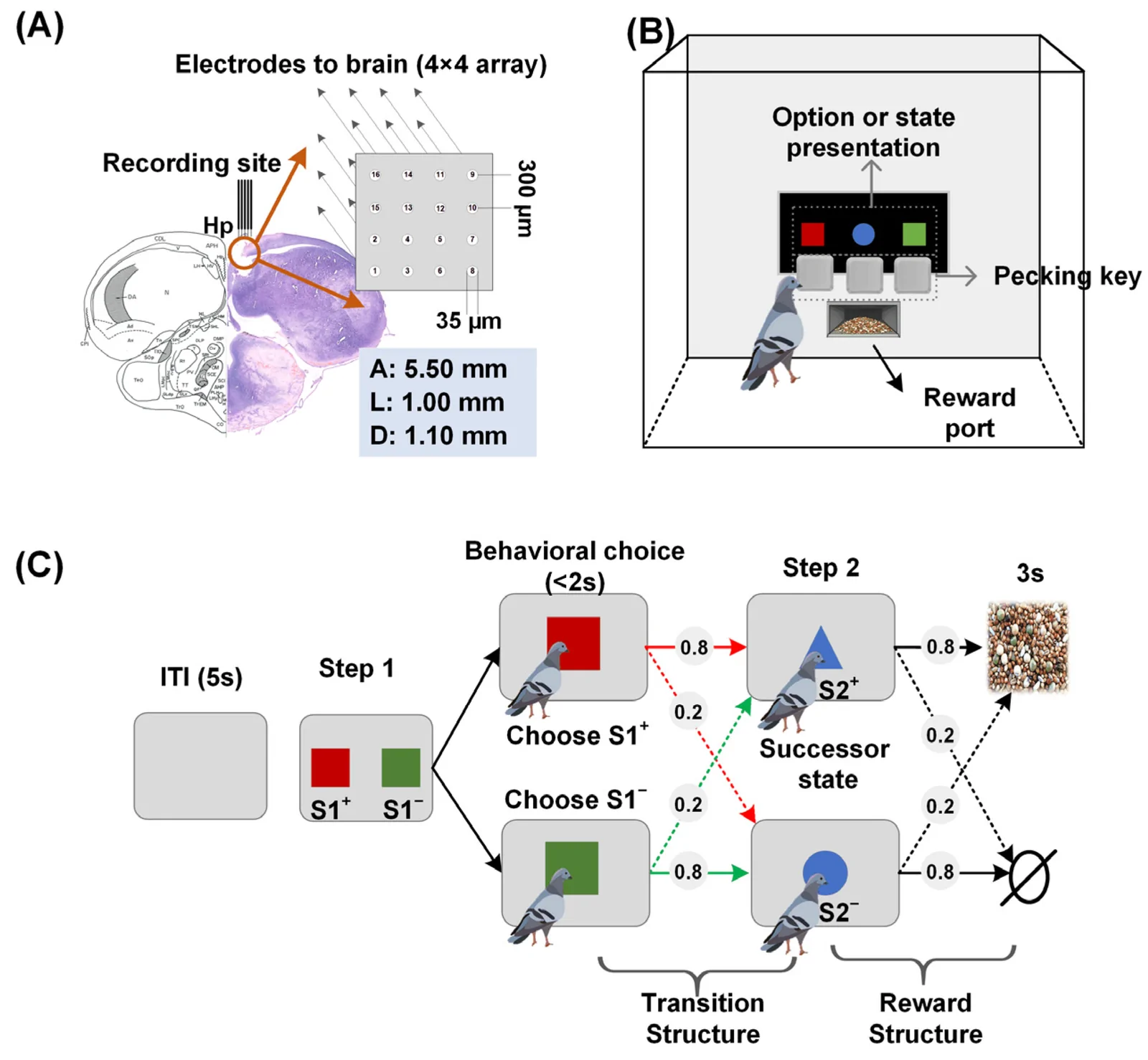
Experimental paradigm, behavioral training apparatus, and brain atlas of the pigeon two-step sequential decision-making task. (**A**) Histological verification of implantation sites. (**B**) Schematic diagram of the pigeon training apparatus. (**C**) The pigeon two-step sequential decision-making task is as follows: a 5-second gray screen indicates that the trial is ready. The first stage (transition structure) is initiated by presenting two distinct color markers (S1^+^ and S1^−^) simultaneously on both sides of the screen. The pigeon indicates its choice by pecking the key below the corresponding target option. Probabilistic transitions occur with probabilities dependent on the pigeon’s choice. Subsequently, a blue triangle (S2^+^) or a circle (S2^−^) marker appears in the center of the screen, indicating the transition outcome and initiating the second stage (reward structure). The pigeon then pecks the key below the S2 target within 2 s. 3-second reward is delivered with the appropriate probability.

Following completion of electrophysiological recordings, histological verification of electrode locations was performed in all animals. Electrolytic marking lesions were generated under deep anesthesia, induced by intramuscular injection of 2% sodium pentobarbital (0.2 mL/100 g body weight) by applying electrical current through the implanted electrodes (1.2 mA, 30 s, repeated three times) to identify the recording sites. The pigeons were subsequently perfused with 4% paraformaldehyde (Henan Celnovte Biotechnology Co., Ltd., Zhengzhou, China), and the brains were removed, frozen, and sectioned into 40 μm coronal slices. Brain sections were processed using Hematoxylin and Eosin (H&E) staining (Henan Celnovte Biotechnology Co., Ltd., Zhengzhou, China). The lesion sites were then compared with the pigeon brain atlas to confirm the anatomical locations of the recording sites (Figure 1A).

### 2.2. Experimental Paradigm and Data Acquisition

Following surgery, pigeons were allowed to recover for one week before behavioral training and electrophysiological recording. Neural signals were acquired using a multichannel acquisition processor (Cerebus™, 128 channels; Blackrock Microsystems, Salt Lake City, UT, USA) at a sampling rate of 2000 Hz. Simultaneous LFP recordings were obtained from 32 channels implanted in the Hp and ST. The recorded signals were low-pass-filtered at 250 Hz using a Butterworth filter. Behavioral events, including stimulus presentation, key peck responses, and reward delivery, were synchronized with electrophysiological recordings throughout the experiment. Pigeons were trained on a two-step sequential decision-making task (Figure 1), which consisted of two consecutive stages: a transition stage (stage 1) and a reward stage (stage 2). The task was designed to separate the effects of state-transition information and reward outcomes during learning. Each experimental session consisted of 150 trials. Training continued for 60 sessions across pigeons until stable acquisition was achieved. Learning stages were defined according to behavioral performance rather than fixed trial numbers. Early learning was defined as periods in which the S1^+^ choice rate was below 65%, whereas stable acquisition was defined as periods in which the S1^+^ choice rate was maintained at ≥85% for at least three consecutive sessions.

At the beginning of each trial, two visual stimuli representing the initial state (S1^+^ and S1^−^) were simultaneously presented on the screen. The stimuli were represented by a red square and a green square, respectively. Pigeons were required to select one stimulus within 2 s by pecking the corresponding response key. Following the choice, the task transitioned to one of two successor states (S2^+^ or S2^−^) according to predefined state-transition probabilities (*T*_S,S’_). Specifically, selecting S1^+^ resulted in a transition to S2^+^ with an 80% probability (common transition) and to S2^−^ with a 20% probability (rare transition). Conversely, selecting S1^−^ resulted in a transition to S2^−^ with an 80% probability and to S2^+^ with a 20% probability.

After the transition, one of the two successor states was presented: S2^+^ (blue triangle) or S2^−^ (blue circle). Pigeons were required to confirm the second-stage state by pecking the corresponding response key within 2 s. Reward delivery was determined by the predefined reward probability (*P*_r_) associated with each successor state. Reaching S2^+^ resulted in food reward delivery (mixed grain) with an 80% probability and no reward with a 20% probability, whereas reaching S2^−^ resulted in reward delivery with a 20% probability and no reward with an 80% probability. When obtained, the reward was delivered for 3 s. After reward or non-reward feedback, a 5-s inter-trial interval (ITI) was initiated before the next trial.

This probabilistic two-step task required the pigeons to simultaneously learn the rules of the environment (how one state transitions to another) and the ultimate payoff (reward information). By using this design, we could investigate how the animals adapt their decision-making strategies over time. Furthermore, it allowed us to examine the underlying ‘neural dynamics’—that is, the moment-to-moment changes in electrical communication and synchronized activity between the Hp and ST as the birds deliberated and executed these sequential choices.

### 2.3. Data Processing

To ensure the reliability of subsequent analyses, behavioral and electrophysiological data were first screened for valid trials. Incomplete trials and trials with abnormal reaction times were excluded, with reaction times in both the first and second stages restricted to 0.5–2.5 s. LFP signals, originally acquired at 2000 Hz, were down-sampled to 1000 Hz before further processing. Correlated noise across recording channels was attenuated using an adaptive common-average reference procedure, and 50 Hz line noise was removed using an adaptive filtering approach. Trials containing prominent movement-related artifacts, such as strong wing flapping, abrupt body movements, or unstable posture, were excluded. In addition, trials showing abnormal high-amplitude LFP fluctuations were rejected when the trial-wise peak-to-peak amplitude exceeded the corresponding session mean by more than five standard deviations. Behavioral events and neural signals were subsequently aligned according to experimental timestamps, and LFP segments were extracted relative to task events. Cross-channel phase transfer entropy (PTE) [29] matrices were then calculated separately for the theta, beta, and broad gamma frequency bands and aggregated according to the sliding-window procedure described below to characterize the temporal evolution of Hp–ST information flow during learning.

### 2.4. Dynamic Reinforcement Learning Modeling of Behavioral Strategy Transitions

To characterize learning-dependent changes in behavioral strategy during the two-step sequential decision-making task, we combined reinforcement-learning (RL) modeling with a sliding-window procedure to track temporal changes in pigeons’ choice behavior [30]. Within each window, MB and MF models were fitted independently and compared using the Bayesian information criterion (BIC). Because MB and MF processes may operate concurrently rather than as mutually exclusive states, we use the terms “MB-like” and “MF-like” to describe their relative statistical support within each window. Specifically, a window was classified as MF-like when the MF model yielded a lower BIC than the MB model, indicating that behavior in that period was better described by value-based learning than by transition-structure-sensitive control. Key computational parameters, including learning rates and inverse temperatures, were also estimated across successive windows to characterize learning-related changes in choice behavior.

The MF model [31] was implemented as a Rescorla–Wagner reinforcement learning model (RW-RL), in which action values were updated based on reward prediction errors. In the second stage (reward stage), the successor-state value $Q_{S2}$ was updated according to the Rescorla–Wagner rule:

$$\delta_{Q_{S2}}\left(i\right)=r\left(i+1\right)-Q_{S2}\left(i\right),$$

(1) $$Q_{S2}\left(i+1\right)\leftarrow Q_{S2}\left(i\right)+\eta \delta_{Q_{S2}}(i),$$

where  r=1 denotes reward obtained, r=0 denotes no reward obtained, and η is the learning rate for the second step.

In Step 1 (state transition stage), RW is used to propagate the successor state value back to update the first step action value $Q_{S1}$:

$$\delta_{Q_{S1}}\left(i\right)=Q_{S2}\left(i\right)+\eta (r\left(i+1\right)-Q_{S2}\left(i\right)-Q_{S1}(i)),$$

(2) $$Q_{S1}\left(i+1\right)\leftarrow Q_{S1}\left(i\right)+\alpha \delta_{Q_{S1}}(i),$$

where α is the learning rate for the first step. The probability of action selection is calculated using the SoftMax strategy:

(3) $$P_{MF}^{j}=\frac{exp(\beta_{MF}\cdot Q_{j}^{MF}(i))}{\sum_{j-1}^{K}exp(\beta_{MF}\cdot Q_{j}^{MF}(i))}$$

where $\beta_{MF}$ is the inverse temperature parameter controlling decision randomness. The full parameter set for the MF model is: $\theta_{MF}=(\alpha ,\beta_{MF},\eta )$.

The MB model was implemented as a transition-based MB reinforcement learning model [32], in which action values were estimated by combining learned state-transition probabilities with expected rewards. The expected reward value of each successor state was defined as:

(4) $$V_{s^{\prime }}=Ε[\gamma Ι(r=1)s_{0}=s^{\prime }]$$

The state transition probability matrix $T_{s,s^{\prime }}$ was estimated from empirical frequencies:

(5) $$T_{s,s^{\prime }}=Ε[\sum_{t\to 0}^{\infty }\gamma^{t}Ι(s_{t}=s^{\prime })|s_{0}=s]$$

where γ is the discount factor representing the importance attached to future rewards.

The state value is jointly calculated by the state transition probability and the reward expectation:

(6) $$Q_{S1}(i)=\sum_{S2}{Τ}_{S1,S2}\cdot V_{S2}(i),$$

The action selection also employs the SoftMax strategy:

(7) $$P_{MB}^{j}=\frac{exp(\beta_{MB}\cdot Q_{j}^{MB}(i))}{\sum exp(\beta_{MB}\cdot Q_{j}^{MB}(i)}$$

The full parameter set for the MB model is: $\theta_{MB}=(\gamma ,\beta_{MB})$.

To capture dynamic changes in behavioral strategy, the models were fitted using a sliding-window procedure with a window size of 200 trials and 50% overlap between adjacent windows. The 200-trial window was selected to balance parameter-estimation stability with temporal resolution. A sufficiently large number of trials is required to obtain reliable estimates of key RL parameters, whereas excessively long windows may obscure gradual changes in strategy across training. Within each window, the parameters of the MB and MF models were estimated independently using maximum a posteriori (MAP) estimation, which combines the likelihood of the observed behavioral data with prior distributions over the model parameters. Weakly informative priors were specified as follows: the learning-rate parameters α and η, as well as the discount factor γ, were assigned Beta(1.1, 1.1) priors, whereas the inverse-temperature parameters $\beta_{MF}$ and $\beta_{MB}$ were assigned Gamma(1.2, 5.0) priors. The parameter set that maximized the posterior probability of the observed choices was selected for each model and fitting window. Model performance was subsequently evaluated using the BIC [33], calculated as $BIC=-2ln\left(L\right)+kln\left(n\right)$, where L is the likelihood of the observed behavioral data under the fitted model, k is the number of free parameters, and n is the number of observations within the fitting window. Lower BIC values indicate better model performance after accounting for model complexity. By comparing the BIC values of the MB and MF models across successive windows, we characterized temporal changes in their relative model support during learning. Model-predicted choice probabilities were further compared with the observed S1^+^ choice rates to assess whether the fitted models captured the major learning-related changes in behavioral performance.

### 2.5. Hp-ST Information Interaction Analysis

To explore the dynamic information interaction characteristics between the Hp and ST during sequential decision-making tasks [34,35], this study used the PTE method to quantify the direction of cross-region information flow and changes in its intensity. Compared to traditional undirected functional connectivity methods, PTE estimates directed information transfer based on phase relationships between oscillatory signals. Considering that cross-regional brain synergy typically relies on specific frequency oscillatory networks, we extracted three core cognitive frequency bands: theta (4–8 Hz), beta (12–30 Hz) and gamma (30–80 Hz). These specific bands were selected based on prior literature indicating their distinct roles in spatial navigation, working memory, and local circuit processing in both avian and mammalian species [36,37,38,39,40]. A broad gamma range was selected to capture the overall high-frequency local circuit coordination commonly associated with cognitive processing in avian models, avoiding pre-emptive subdivision into low and high gamma bands which are less strictly demarcated in the pigeon brain. By calculating the directed information flow weights for each band, we aimed to comprehensively evaluate the directional bias of the Hp-ST circuit information flow and its dynamic evolution across sliding time windows during learning.

To investigate the dynamic information interactions between the Hp and ST during the sequential decision-making task (specifically, the red cue selection phase), we first designated the timestamp of the first key-peck action in each trial as time zero. We then extracted the LFP signals for 0.5 s prior to the action (i.e., 0.5 s to 0 s relative to the action onset) as the analytical time window representing the decision-preparation phase. This 500 ms pre-motor window was chosen because it captures the critical period of deliberation and model-based valuation immediately preceding the behavioral commitment, avoiding post-motor execution artifacts. Subsequently, a fourth-order Butterworth band-pass filter was applied to decompose the signals into the three classic cognitive bands (Theta, Beta and Gamma). The instantaneous phase of each channel’s signal was extracted via the Hilbert transform. For a given frequency-band band-pass-filtered LFP signal x(t), its corresponding analytical signal z(t) is constructed by:

(8) z(t)=x(t)+iH[x(t)]

where H[x(t)] is the Hilbert transform of the original signal x(t). The instantaneous phase ϕ(t) at time t is computed as:

(9) ϕ(t)=arctan((H[x(t)])/(x(t)))

Transfer entropy is built upon conditional mutual information [41,42,43]. Let X denote the phase time series of the source channel and Y denote the phase time series of the target channel. Given a prediction delay time τ, the PTE from X to Y, denoted as ${PTE}_{X\to Y}$, is defined as the reduction in uncertainty about the future phase of Y achieved by introducing the past phase of X. Using Bayes’ theorem, this expands into joint probability densities:

(10) $${PTE}_{X\to Y}=\sum p(Y_{t+\tau },Y_{t},X_{t})\log\frac{p(Y_{t+\tau },Y_{t},X_{t})\cdot p(Y_{t})}{p(Y_{t+\tau },Y_{t})\cdot p(Y_{t},X_{t})}$$

In the PTE calculations, the delay time was set to 5 ms.

To capture dynamic neural reorganization during learning, we employed a sliding window approach with a window size of 25 consecutive valid trials. Within each window, we computed the average PTE intensity across all channels for Hp→ST and ST→Hp separately, and calculated the net information flow (ΔPTE):

(11) $$\Delta PTE=\bar{{PTE}_{Hp\to ST}}-\bar{{PTE}_{ST\to }}$$

A value of ΔPTE>0 indicates that the Hp dominates network information sending, whereas ΔPTE<0 implies ST dominance.

To evaluate the overall significance of brain region interactions throughout the task, we utilized the non-parametric Wilcoxon signed-rank test to compare the average information flow intensities of Hp→ST and ST→Hp across all sliding windows. The significance level was set at alpha = 0.05.

### 2.6. Association Between Behavioral Model Evidence and Gamma-Band Information Flow

To examine the relationship between behavioral strategy dynamics and gamma-band Hp-to-ST information flow, a relative model-evidence index was calculated as $\Delta BIC=BIC_{MF}-BIC_{MB}$. Because behavioral-model and neural-information-flow analyses used different window sizes, their trajectories were independently normalized from 0 to 1 for each pigeon and divided into five equivalent learning-progress bins. Mean ΔBIC and gamma-band Hp-to-ST PTE were calculated within each bin and standardized within pigeon. Within-pigeon correlations were used to characterize their overall correspondence, followed by partial-correlation analyses controlling for normalized learning progression. Stage 1 and Stage 2 were analyzed separately. Models additionally including a quadratic learning-progress term were examined as sensitivity analyses.

### 2.7. Statistical Analysis

To characterize dynamic changes in Hp–ST information flow during learning, statistical analyses were performed at both the sliding-window and animal levels. Within each sliding window, PTE values representing Hp→ST and ST→Hp information transfer were extracted separately for each frequency band. The Wilcoxon signed-rank test was used to assess directional asymmetry between Hp→ST and ST→Hp information flow, with statistical significance defined as *p* < 0.05.

To evaluate whether the directional preference of Hp–ST information flow was consistently expressed across animals, net information flow (Hp→ST minus ST→Hp) was calculated separately for each pigeon, frequency band, and task stage. These values were summarized across sliding windows to characterize the overall direction and magnitude of Hp–ST information transfer at the individual-animal level.

Learning-related changes in directed information flow were assessed using ordinary least-squares linear regression separately for each pigeon. The sliding-window index was entered as the independent variable, and Hp→ST PTE strength was entered as the dependent variable. The coefficient of determination (*R*^2^), regression slope, corresponding *p*-value, and 95% confidence interval were used to characterize the magnitude and temporal direction of the learning-related effect. Because gamma-band Hp→ST information flow represented the principal frequency-specific finding, the eight gamma-band regression tests obtained from four pigeons across the two task stages were additionally corrected for multiple comparisons using the Benjamini–Hochberg false discovery rate (FDR) procedure. An FDR-adjusted *q* < 0.05 was considered statistically significant. Both raw *p*-values and FDR-adjusted *q*-values are reported for these analyses.

Given the limited number of animals, emphasis was placed on within-animal longitudinal effects and the consistency of results across individual pigeons rather than inference based solely on group averages. All statistical analyses were performed using MATLAB 2023a, GraphPad Prism 10.1.2, and OriginPro 2019 Learning Edition.

## 3. Results

### 3.1. Behavioral Performance in the Pigeon Two-Step Sequential Decision-Making Task

**Figure 2:**
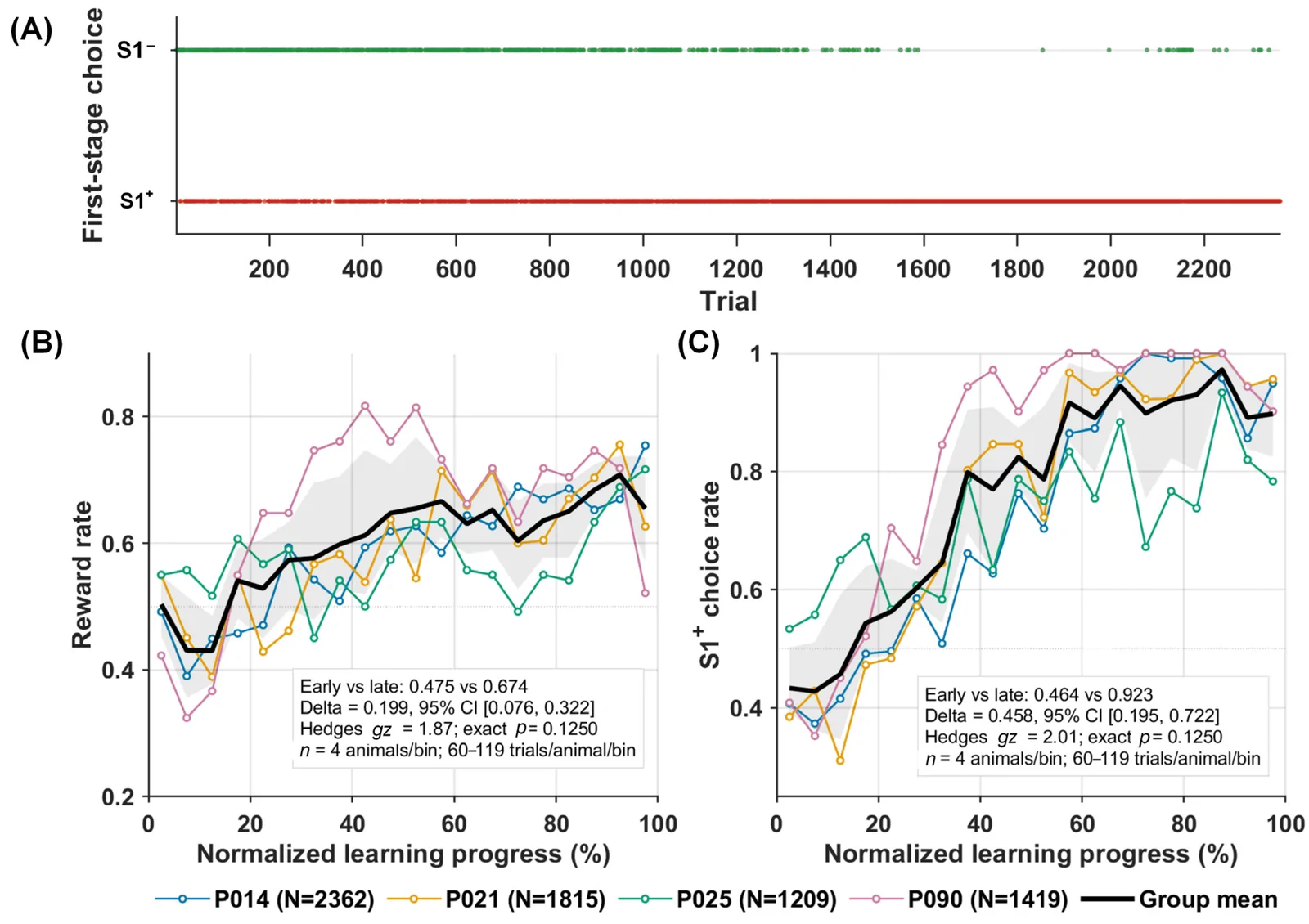
Individual and group-level behavioral changes during acquisition of the two-step sequential decision-making task. (**A**) Trial-by-trial first-stage choices from a representative pigeon (P014), illustrating a gradual transition from variable selection between the nonoptimal option and the optimal option S1^+^ to stable selection of S1^+^ during later learning. (**B**,**C**) Individual and group trajectories of reward rate (**B**) and S1^+^ choice rate (**C**). To account for differences in training duration, each pigeon’s trial sequence was normalized from 0% to 100% and divided into 20 non-overlapping bins, each representing 5% of the total trials. Colored lines represent individual pigeons (P014, P021, P025, and P090), the thick black line represents the group mean, and the gray shaded area indicates the 95% bootstrap confidence interval across pigeons. The horizontal dotted line indicates chance level (0.5). Numbers in parentheses in the legend indicate the total number of trials contributed by each pigeon; each normalized bin contained 60–119 trials per pigeon, with all four pigeons contributing to every bin. Statistical annotations compare the early (0–20%) and late (80–100%) learning phases using an exact two-sided paired sign-flip test. Reward rate increased from 0.475 to 0.674 (late-early difference, Δ = 0.199, 95% CI [0.076, 0.322], Hedges’ *gz* = 1.87, exact *p* = 0.125), while S1^+^ choice rate increased from 0.464 to 0.923 (Δ = 0.458, 95% CI [0.195, 0.722], Hedges’ *gz* = 2.01, exact *p* = 0.125; *n* = 4 pigeons).

Before reinforcement-learning modeling, we first examined behavioral performance during task acquisition. As illustrated in Figure 2A, the representative pigeon P014 gradually shifted from variable first-stage choices toward increasingly stable selection of the optimal S1^+^ option as training progressed. To characterize learning-related behavioral changes across all individuals while accounting for differences in training duration, each pigeon’s trial sequence was normalized from 0% to 100% of learning progress and divided into 20 non-overlapping bins. Individual trajectories of both reward rate and S1^+^ choice rate showed an overall upward trend across learning (Figure 2B,C), although the magnitude and temporal profile of these changes varied among pigeons.

To quantify the overall behavioral improvement, we compared the early (0–20%) and late (80–100%) phases of normalized learning progress. The mean reward rate increased from 0.475 during the early phase to 0.674 during the late phase (Δ = 0.199, 95% CI [0.076, 0.322], Hedges’ *gz* = 1.87, exact *p* = 0.125). Similarly, the mean S1^+^ choice rate increased from 0.464 to 0.923 (Δ = 0.458, 95% CI [0.195, 0.722], Hedges’ *gz* = 2.01, exact *p* = 0.125). Although these paired comparisons did not reach the conventional significance threshold, both measures showed large effect sizes and consistent learning-related increases across the four pigeons. Together, these results indicate that task acquisition was accompanied by a marked improvement in reward attainment and a progressive stabilization of optimal first-stage choice behavior.

### 3.2. Dynamic Behavioral Strategy Shifts Revealed by Reinforcement Learning Modeling

**Figure 3:**
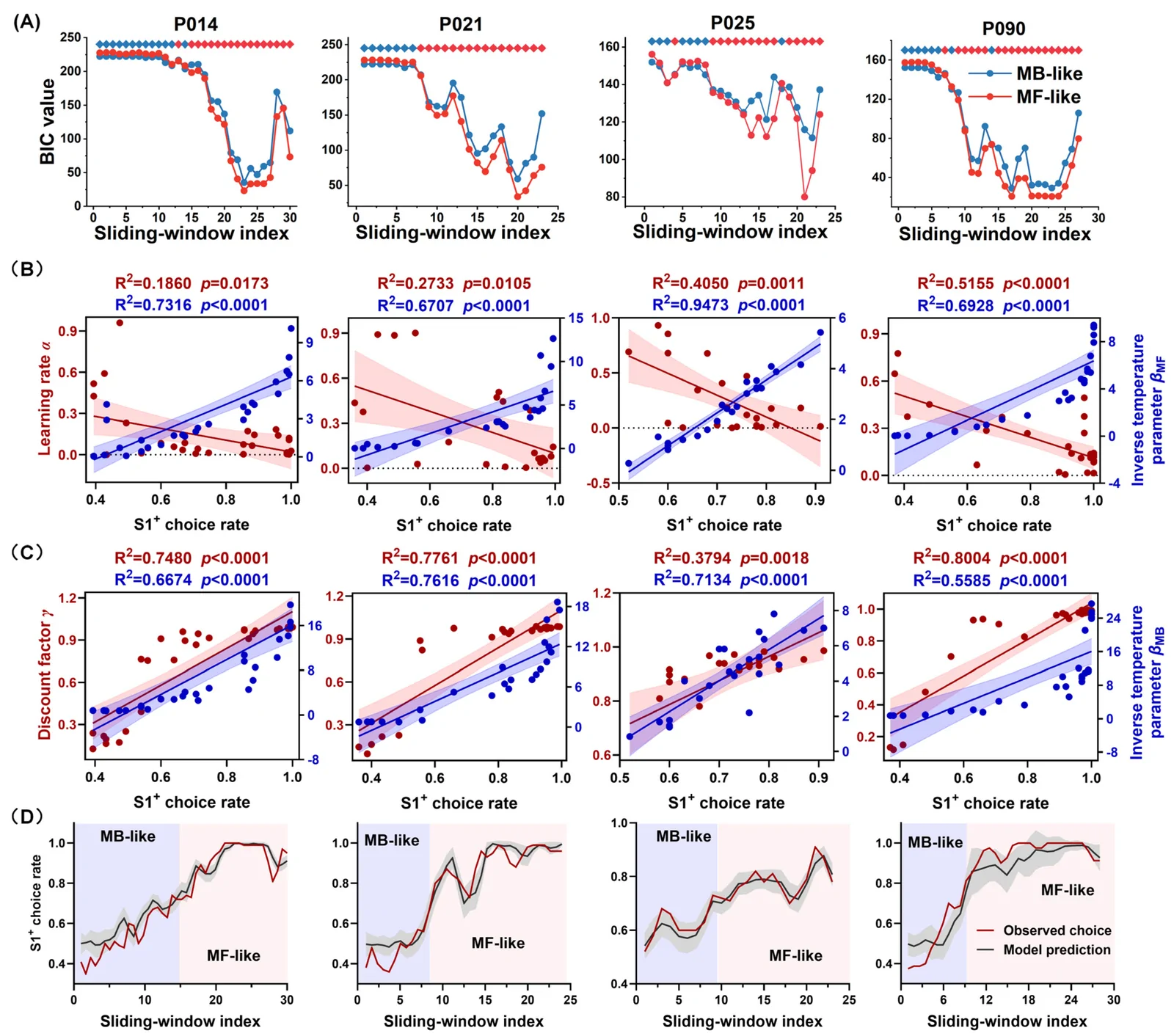
Dynamic reinforcement learning modeling of behavioral strategy changes during task acquisition. (**A**) Dynamic comparison of model fit across successive sliding windows for four pigeons (P014, P021, P025, and P090). BIC values are shown for the MB-like and MF-like models, with lower BIC indicating better model fit. The MB-like model generally provided a better account of behavior during early learning, whereas the MF-like model became more favorable during later learning. (**B**) Relationships between MF-model parameters and the S1^+^ choice rate. The learning rate (red, left axis) decreased as the S1^+^ choice rate increased, whereas the inverse temperature parameter (blue, right axis) increased, indicating reduced trial-by-trial updating and increasingly stable value-guided choice as learning progressed. (**C**) Relationships between MB-model parameters and the S1^+^ choice rate. Both the discount factor (red, left axis) and inverse temperature parameter (blue, right axis) showed systematic increases with improving task performance. Solid lines indicate linear regression fits, shaded areas represent 95% confidence intervals, R^2^ and *p*-values are shown above each panel. (**D**) Comparison between the observed S1^+^ choice rate and model predictions across sliding windows. The close correspondence between observed and predicted behavioral trajectories indicates that the dynamic RL models captured the major changes in choice behavior during learning. Shaded background regions indicate the learning periods in which behavior was better characterized as MB-like or MF-like.

To further characterize the evolution of behavioral strategies during learning, dynamic reinforcement learning models were fitted across successive sliding windows. As shown in Figure 3A, the MB-like model generally yielded lower BIC values during early learning, whereas the MF-like model provided a better fit during later learning. Although the timing of this transition varied across individuals, a broadly consistent shift in model preference was observed across the four pigeons. These results suggest that behavior was initially better explained by a strategy sensitive to the task transition structure, whereas later choices were increasingly captured by value-based MF-like control as behavioral performance stabilized.

The dynamic changes in model parameters further characterized the learning-related adjustment of decision strategies. For the MF model (Figure 3B), the learning rate decreased whereas the inverse temperature increased with increasing S1^+^ choice rate, indicating reduced sensitivity to individual outcomes and increasingly stable exploitation of learned action values. For the MB model (Figure 3C), both the discount factor and inverse temperature increased with behavioral performance, suggesting greater weighting of future outcomes and more consistent model-based choice. Overall, these parameter changes indicate that learning was accompanied by a gradual reduction in behavioral variability and increasingly stable value-guided decision-making.

Finally, the model-predicted S1^+^ choice rates closely followed the observed behavioral trajectories across successive sliding windows (Figure 3D), indicating that the dynamic RL models captured the major learning-related changes in choice behavior. Taken together, these results reveal a learning-dependent reorganization of behavioral strategy, characterized by a gradual shift from early MB-like, task-structure-sensitive control toward later MF-like, stable value-guided choice.

### 3.3. Predominant Hp-to-ST Directed Information Flow Across Both Task Stages

**Figure 4:**
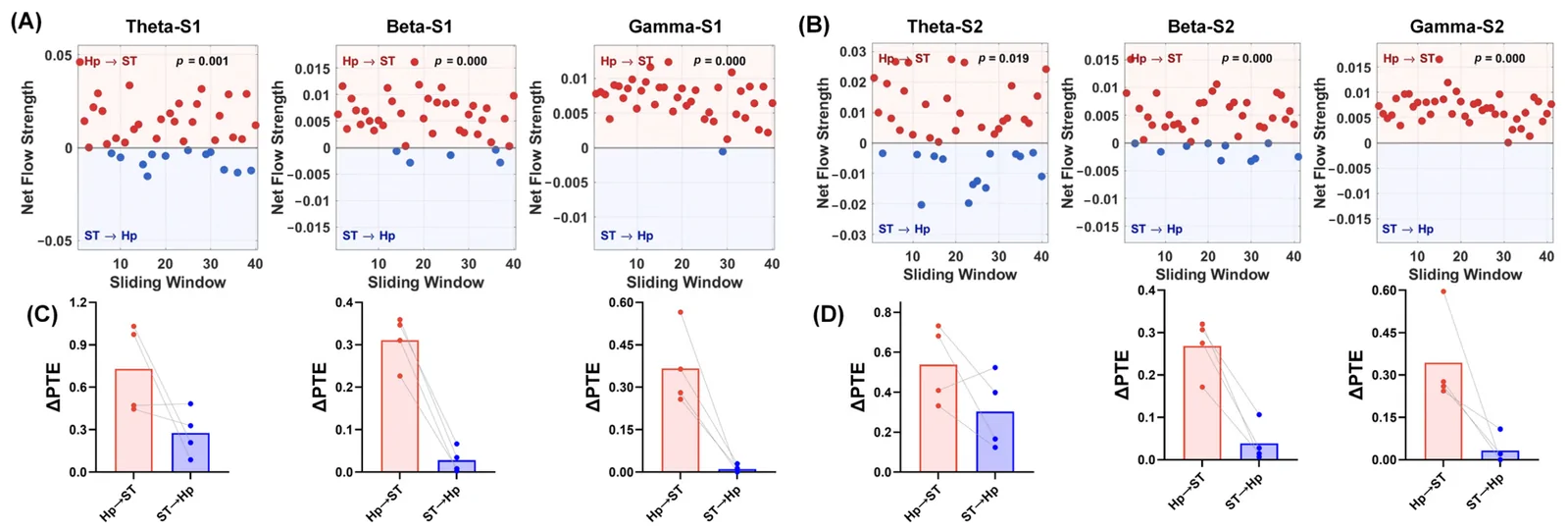
Predominant Hp-to-ST directed information flow across the two stages of sequential decision-making. (**A**,**B**) Dynamic net information flow between the Hp and ST across successive sliding windows during Stage 1 (**A**) and Stage 2 (**B**). Results are shown separately for the theta, beta, and gamma bands. Positive values (red) indicate predominant Hp→ST information flow, whereas negative values (blue) indicate predominant ST→Hp information flow. The *p*-values indicate comparisons between Hp→ST and ST→Hp information flow across sliding windows using the Wilcoxon signed-rank test. (**C**,**D**) Individual-animal comparison of Hp→ST and ST→Hp information flow across four pigeons during Stage 1 (**C**) and Stage 2 (**D**). Each dot represents one pigeon, and paired points from the same animal are connected by gray lines. Bars represent the mean across animals. Across all three frequency bands and both task stages, all four pigeons exhibited greater Hp→ST than ST→Hp information flow, demonstrating a consistent directional bias across individuals.

To characterize the directional organization of Hp–ST communication during sequential decision-making, we quantified net information flow between the Hp and ST using PTE across the theta, beta, and gamma bands. It is important to emphasize that PTE provides an estimate of directed statistical forecasting rather than a direct measure of anatomical connectivity or causal neural influence. Positive net flow indicates predominant Hp→ST information transfer, whereas negative values indicate predominant ST→Hp information transfer. As shown in Figure 4A, B, a clear directional asymmetry was observed across successive sliding windows in both task stages. During Stage 1 (Figure 4A), net information flow was predominantly directed from the Hp to the ST across the theta, beta, and gamma bands, with Hp→ST flow significantly exceeding the reverse direction (theta: *p* = 0.001; beta and gamma: *p* < 0.001). A similar pattern was observed during Stage 2 (Figure 4B), where Hp→ST information flow remained predominant across all three frequency bands (theta: *p* = 0.019; beta and gamma: *p* < 0.001). Although occasional windows exhibited ST→Hp-dominant flow, the overall directional organization consistently favored Hp→ST communication. The consistency of this directional bias across animals was further examined in four pigeons (Figure 4C,D). In both Stage 1 and Stage 2, all four pigeons showed greater Hp→ST than ST→Hp information flow across the theta, beta, and gamma bands, although the magnitude of the directional difference varied among individuals. Thus, the Hp→ST predominance observed across sliding windows was also consistently expressed at the individual-animal level.

Together, these results indicate that Hp–ST communication exhibits a robust directional asymmetry during sequential decision-making, with information flow predominantly organized from the Hp toward the ST across both task stages and multiple frequency bands. This consistent Hp→ST directional organization provides the basis for examining how its strength is dynamically reorganized as learning progresses.

### 3.4. Gamma-Band Attenuation of Hp-to-ST Information Flow During Learning

**Figure 5:**
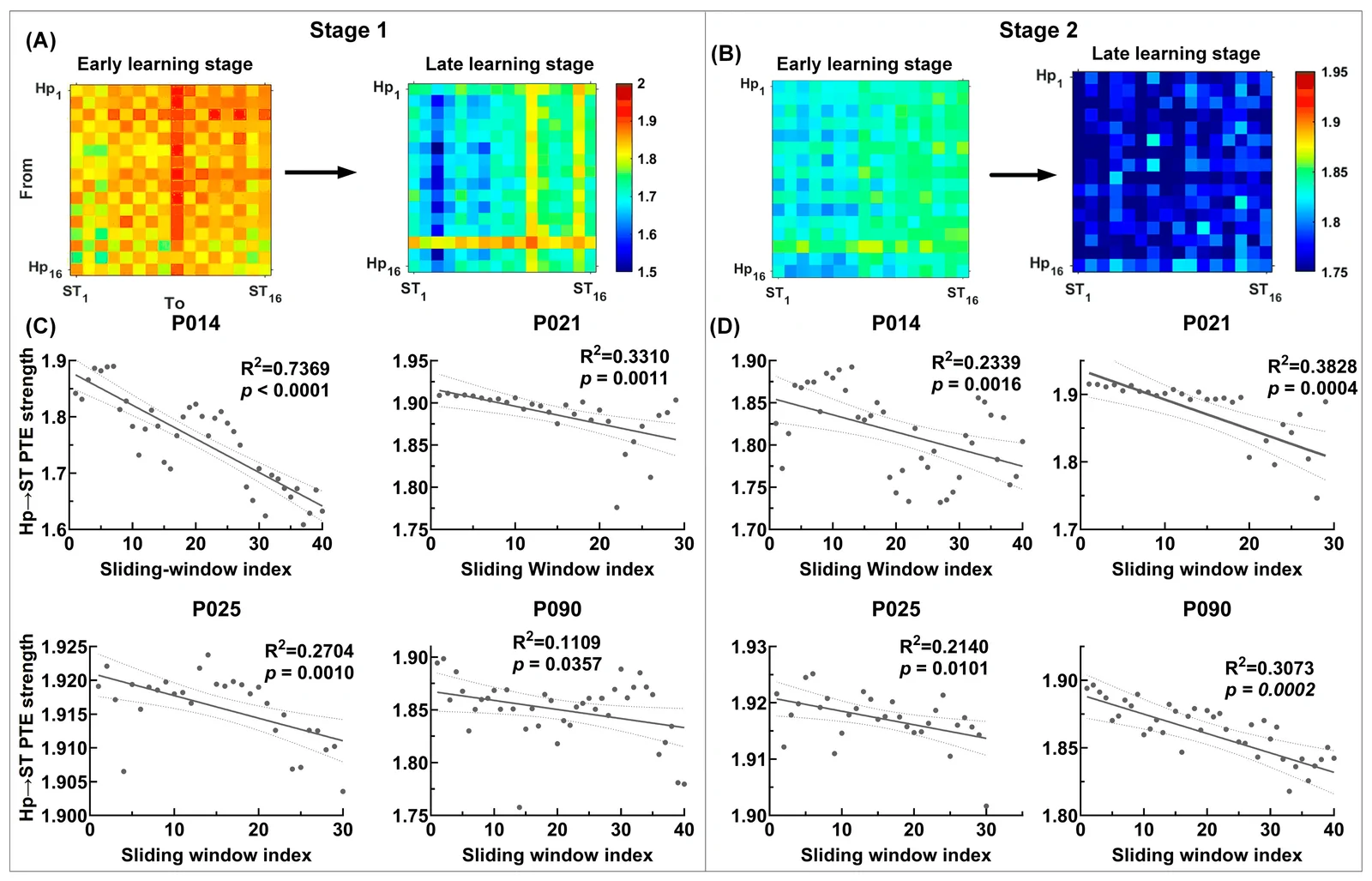
Learning-related attenuation of Hp-to-ST gamma-band directed information flow. (**A**,**B**) Representative early- and late-learning Hp→ST gamma-band PTE matrices from pigeon P014 during Stage 1 (**A**) and Stage 2 (**B**). Matrices represent directed information flow from 16 Hp channels (Hp_1_–Hp_16_) to 16 ST channels (ST_1_–ST_16_). Color intensity represents Hp→ST gamma-band PTE strength. Compared with early learning, late-learning matrices show reduced directed interaction strength in both task stages. (**C**,**D**) Linear regression analyses of Hp→ST gamma-band PTE strength across successive sliding windows for four pigeons during Stage 1 (**C**) and Stage 2 (**D**). Each point represents the mean Hp→ST gamma-band PTE strength within an individual sliding window. Solid lines indicate linear regression fits, and dashed lines indicate 95% confidence intervals. *R^2^* and *p*-values are shown for each individual pigeon. All pigeons exhibited significant negative relationships between learning progression and gamma-band Hp→ST PTE strength, demonstrating a consistent attenuation of gamma-band directed communication during learning.

Following the identification of a predominant Hp-to-ST directional bias across task stages, we further investigated whether the strength of this directed interaction changed throughout learning. Because gamma-band communication showed the strongest learning-related modulation, we analyzed the temporal evolution of Hp→ST gamma-band PTE strength across successive sliding windows. As shown in Figure 5A,B, representative Hp→ST gamma-band PTE matrices from pigeon P014 exhibited a clear reduction from early to late learning in both Stage 1 and Stage 2, indicating a progressive attenuation of gamma-band directed interaction as task performance became more stable.

To quantify this learning-related change, linear regression analyses were performed separately for each pigeon, using the sliding-window index as the independent variable and Hp→ST gamma-band PTE strength as the dependent variable (Figure 5C,D). During Stage 1, each pigeon showed a significant negative association between learning progression and gamma-band Hp→ST PTE strength (P014: *R*^2^ = 0.7369, *p* < 0.0001; P021: *R*^2^ = 0.3310, *p* = 0.0011; P025: *R*^2^ = 0.2704, *p* = 0.0010; P090: *R*^2^ = 0.1109, *p* = 0.0357). A similar pattern was observed during Stage 2 (P014: *R*^2^ = 0.2339, *p* = 0.0016; P021: *R*^2^ = 0.3828, *p* = 0.0004; P025: *R*^2^ = 0.2140, *p* = 0.0101; P090: *R*^2^ = 0.3073, *p* = 0.0002). To account for multiple testing across the four pigeons and two task stages, the eight regression *p*-values were corrected using the Benjamini–Hochberg FDR procedure. All effects remained significant after correction, with FDR-adjusted *q*-values ranging from 0.0008 to 0.0357 (Table 1), supporting the robustness of the learning-related gamma-band attenuation.

In contrast, the temporal dynamics of Hp→ST information flow in the theta and beta bands were less consistent across animals (Table 2). Significant learning-related changes were observed only in some individuals, whereas others showed no clear temporal trend. This contrast indicates that the learning-related reorganization of Hp–ST communication was frequency-specific, with gamma-band directed information flow showing the most consistent attenuation across individuals and task stages. Together, these findings show that learning progression was accompanied by a selective and statistically robust reduction in gamma-band Hp→ST directed information flow while the overall Hp→ST directional organization remained preserved. This pattern suggests that gamma-band Hp–ST interaction is particularly sensitive to changes in learning state and may represent a neural correlate of the transition toward more stable task performance.

### 3.5. Relationship Between Behavioral Model Evidence and Gamma-Band Hp-to-ST Information Flow

To directly examine whether the learning-related reduction in gamma-band Hp-to-ST information flow was associated with the inferred behavioral strategy transition, we calculated a continuous relative model-evidence index as $\Delta BIC=BIC_{MF}-BIC_{MB}$, with positive values indicating relatively greater support for the MB-like model. Because the behavioral and neural analyses used different sliding-window sizes, the two trajectories were independently normalized from 0 to 1 for each pigeon and divided into five equivalent learning-progress bins. Mean ΔBIC and gamma-band Hp-to-ST PTE were calculated within each bin and standardized within pigeon.

**Figure 6:**
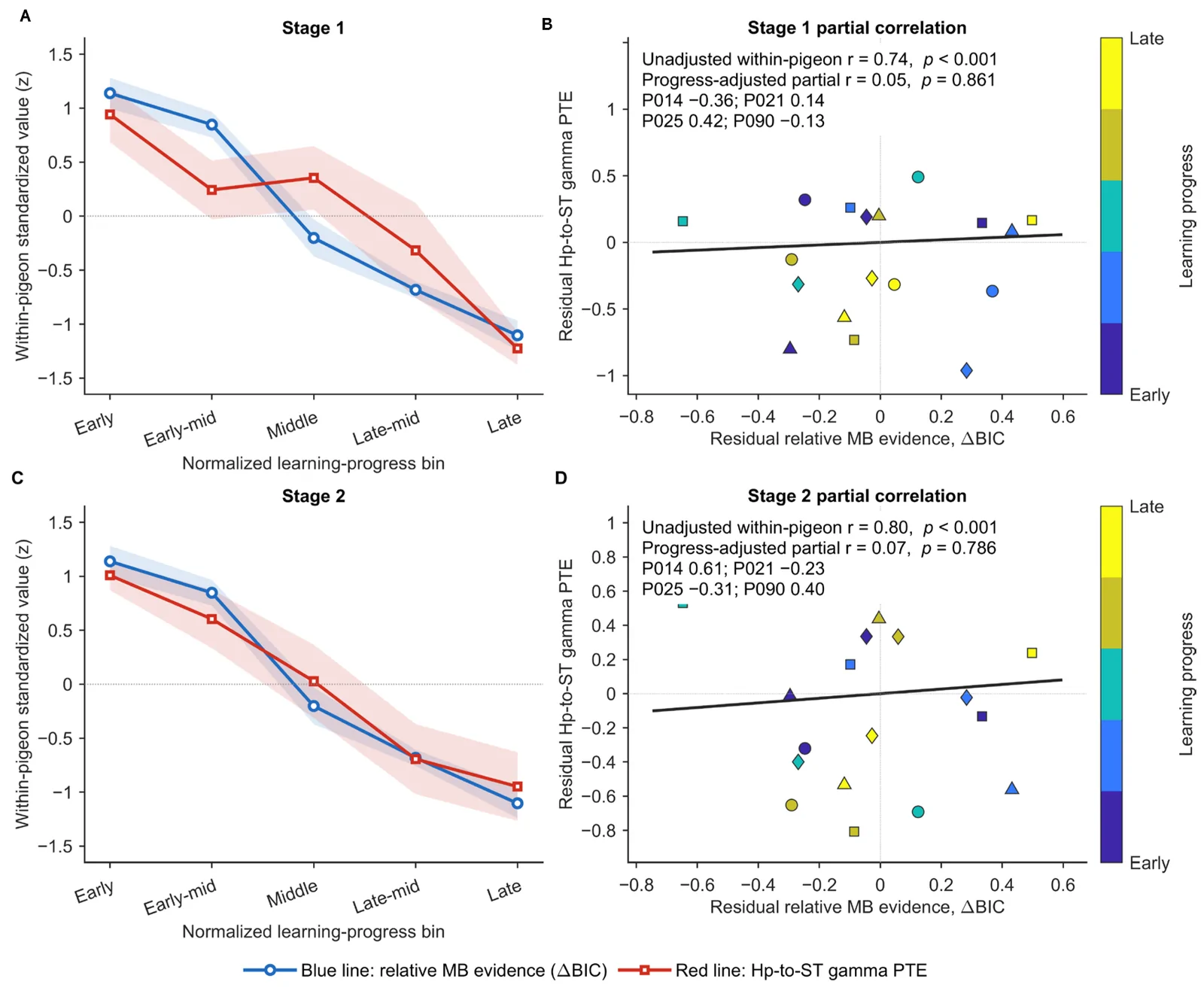
Learning-progress-level correspondence between behavioral model evidence and gamma-band Hp-to-ST information flow. Behavioral and neural trajectories were independently normalized for each pigeon and divided into five equally spaced learning-progress bins. Analyses included four pigeons (P014, P021, P025, and P090). Relative MB evidence was quantified as $\Delta BIC=BIC_{MF}-BIC_{MB}$, with positive values indicating greater relative support for the MB-like model. All measures were standardized within pigeon. (**A**,**C**) Mean ± SEM trajectories of ΔBIC and gamma-band Hp-to-ST PTE during Stage 1 and Stage 2. (**B**,**D**) Partial-correlation plots after removing pigeon effects and linear learning progression. Marker shape identifies pigeon, and color denotes learning progression from early to late. Significant unadjusted within-pigeon associations were observed in both stages, whereas the progress-adjusted partial correlations were not significant.

As shown in Figure 6A,C, relative MB model evidence and gamma-band Hp-to-ST PTE exhibited broadly corresponding decreases across learning progression. Before controlling for learning progression, significant positive within-pigeon associations were observed during both Stage 1 (r=0.745, p<0.001) and Stage 2 (r=0.796,p<0.001). Thus, learning periods characterized by relatively greater MB-like model evidence also tended to show stronger gamma-band Hp-to-ST information flow.

However, after controlling for normalized learning progression, the associations were no longer significant in either Stage 1 (partial r=0.048, p=0.861; Figure 6B) or Stage 2 (partial r=0.074, p=0.786; Figure 6D). Individual-pigeon partial correlations were also heterogeneous and non-significant. A sensitivity analysis additionally controlling for a quadratic learning-progress term yielded the same conclusion. These findings indicate that behavioral model evidence and gamma-band Hp-to-ST information flow exhibit parallel learning-related trajectories, but the present data do not demonstrate an association independent of their shared relationship with training progression.

## 4. Discussion

This study combined dynamic reinforcement-learning modeling with frequency-specific PTE analysis to examine learning-related changes in Hp–ST communication during sequential decision-making in pigeons. Behavioral modeling revealed a gradual shift from early MB-like, task-structure-sensitive control toward later MF-like value-guided behavior. Across theta, beta, and broad gamma bands, Hp→ST information flow showed a consistent directional bias, while gamma-band Hp→ST flow decreased most consistently with learning. Relative MB model evidence and gamma-band PTE also changed in parallel, but their association was no longer significant after controlling for learning progression. Together, these preliminary findings indicate that behavioral strategy adjustment and Hp–ST communication undergo coordinated, but not necessarily directly coupled, changes during sequential learning.

The behavioral transition observed here is broadly consistent with the dual-system framework of reinforcement learning [44,45]. MB control supports flexible decisions by incorporating knowledge of task structure, whereas MF control relies more strongly on values accumulated from previous outcomes to guide efficient choices. In rodents, the hippocampus has been implicated in MB planning and prospective evaluation [46], while computational accounts suggest that Hp and ST systems can jointly support decision-making by integrating state representations with value-based action selection [47]. In the present study, the early advantage of the MB-like model suggests greater sensitivity to transition information during initial learning, whereas the later advantage of the MF-like model indicates an increasing contribution of learned value information as behavior stabilized. Importantly, MB and MF processes should not be regarded as mutually exclusive states. Moreover, because the present task did not include outcome devaluation or contingency degradation procedures, the later behavioral pattern is more appropriately interpreted as MF-like value-guided performance rather than definitive habit formation.

At the neural level, the observed Hp→ST directional bias suggests an asymmetric organization of Hp–ST interactions during sequential decision-making. The Hp contributes to representing environmental states and contextual relationships, whereas the ST integrates reward-related information and supports action selection. Previous studies have emphasized complementary contributions of the Hp–ST axis to learning, prediction, and goal-directed behavior [48]. Theta oscillations have also been implicated in the temporal organization of hippocampal processing during learning [49]. From a comparative perspective, birds possess sophisticated cognitive abilities supported by highly organized pallial and subpallial networks [50,51]. More broadly, hippocampal contributions to value-based decisions have also been demonstrated in mammalian studies [52]. The present findings extend this framework by demonstrating a reproducible Hp→ST directional preference in an avian sequential-learning task. Nevertheless, because PTE reflects directed statistical dependence rather than direct anatomical transmission or causal influence, this directional asymmetry should be interpreted as a property of the recorded neural signals rather than evidence that the Hp causally controls the ST.

A major finding of the present study is the frequency-specific attenuation of broad gamma-band (30–80 Hz) Hp→ST information flow during learning. Gamma oscillations across this broad spectrum have been associated with local circuit processing, feature integration, and selective communication between neuronal populations [53]. Therefore, the stronger gamma-band Hp→ST interaction observed during early learning may reflect increased coordination demands when pigeons were acquiring task structures and updating behavioral values. As task performance became more stable, reduced gamma-band information flow may instead reflect altered coordination requirements or a different organization of Hp–ST communication. This interpretation is consistent with previous pigeon studies showing that high-frequency oscillatory activity and network connectivity are associated with demanding spatial learning and behavioral adjustment. Importantly, reduced PTE should not be interpreted as diminished functional importance of either region, nor can the present data determine whether this reduction reflects greater neural efficiency. 

The frequency specificity of Hp–ST reorganization further suggests that different oscillatory channels may support distinct aspects of sequential learning. Although Hp→ST directional bias was observed across theta, beta, and gamma bands, only gamma-band interactions exhibited a consistent attenuation across individuals. Theta oscillations are generally associated with temporal organization, spatial navigation, and long-range coordination, whereas beta activity has been linked to the maintenance of ongoing cognitive or motor states [54]. Gamma-band interactions may therefore be particularly sensitive to changes in local and interregional processing demands as learning progresses. In this sense, gamma-band attenuation may serve as a neural correlate of increasingly stable task performance, but the present data do not establish that it specifically encodes the transition from MB-like to MF-like control.

The comparative implications of these findings should also be considered in light of differences between avian and mammalian brain organization. In mammals, Hp–ST interactions have been implicated in linking spatial and contextual representations with action selection across learning [55], and both systems contribute to the organization of navigation and value-guided behavior [56]. Avian Hp and basal ganglia systems may perform broadly analogous computational functions, but their anatomical organization and projection patterns are not identical to those of mammals. Previous studies in pigeons have reported enhanced Hp–NCL interactions during visual–spatial associative learning [57] and frequency-specific hippocampal network reorganization during spatial path adjustment [58]. Related studies have also implicated NCL activity and gamma-band oscillations in goal-directed navigation and decision-making [59,60]. The present findings add Hp–ST directional dynamics to this comparative framework and suggest that interactions between environmental representations and value-guided action systems may represent a broadly shared computational motif across vertebrates, without implying identical circuitry. At a broader physiological level, these cross-species parallels may help frame general questions about how state-representation and value-based action systems interact during adaptive decision-making, including in mammals and humans. Such learning-dependent neural dynamics may also provide biologically grounded hypotheses for brain-inspired adaptive decision algorithms, although these translational implications remain speculative.

Several alternative explanations for the gamma-band attenuation should be considered. Learning is accompanied not only by changes in decision strategy but also by changes in attention, novelty, uncertainty, motivational state, motor automatization, and reward prediction. The RL results provide information about learning-related changes in value updating and choice consistency, while the exclusion of incomplete trials and responses outside the predefined reaction-time range reduces the influence of gross variations in task engagement. Nevertheless, the present dataset cannot fully dissociate strategy change from habituation, attentional changes, motivational variation, or increasing motor automatization. Importantly, the additional behavioral–neural analysis showed that relative MB model evidence and gamma-band Hp→ST PTE were positively associated across learning, but this relationship disappeared after controlling for learning progression. Thus, the two measures exhibit parallel temporal trajectories, but the current data do not support an independent strategy-specific relationship between them. The gamma-band effect should therefore be interpreted as accompanying the broader learning process rather than as a direct neural marker or mediator of the MB-like to MF-like transition. Future studies employing reward devaluation, contingency degradation, reversal learning, or additional attentional manipulations will be required to distinguish these overlapping behavioral and cognitive processes.

Several considerations define the scope within which the present findings should be interpreted. Because our analyses were based on LFP-derived PTE between the Hp and ST, the observed directional asymmetry reflects statistical dependence between neural signals rather than direct anatomical connectivity or causal influence. Common input, volume conduction, differences in signal-to-noise ratio, and other unmeasured factors may also influence PTE estimates. Surrogate-based validation was not performed in the present study and would provide a useful complementary control in future analyses. Moreover, the Hp and ST represent only two components of a much broader distributed network involved in learning and decision-making, and the present region-focused approach therefore provides only a partial view of brain-wide coordination. The limited sample of four pigeons further restricts generalization across individuals, despite the extensive longitudinal observations obtained from each animal, and the present findings should consequently be regarded as preliminary. Likewise, the MB-like and MF-like strategies identified here represent computational descriptions inferred from model fitting rather than directly observed cognitive states. Future studies with larger cohorts should combine more temporally resolved computational modeling, complementary connectivity measures, and reversible modulation of Hp or ST activity during different learning phases to more directly test the functional contribution of these interactions. Extending the paradigm to tasks with different spatial, mnemonic, transition, or reward contingencies will also help determine whether the observed gamma-band attenuation represents a general feature of learning-related Hp–ST reorganization or a task-specific phenomenon. Together, such approaches will help clarify how local Hp–ST interactions are embedded within the broader neural systems that support adaptive decision-making.

## 5. Conclusions

In conclusion, this preliminary study shows that sequential learning in pigeons is accompanied by coordinated changes in behavioral strategy and Hp–ST information flow. Behavioral modeling revealed a gradual shift from early MB-like, task-structure-sensitive control toward later MF-like value-guided behavior, while Hp→ST information flow showed a consistent directional bias across theta, beta, and gamma bands. Notably, gamma-band Hp→ST information flow progressively decreased as learning advanced, indicating frequency-specific reorganization of Hp–ST communication. However, these findings are based on only four pigeons and on LFP-derived PTE, which reflects directed statistical dependence rather than causal or anatomical connectivity. Moreover, the Hp and ST represent only part of the broader distributed neural systems supporting learning and decision-making. Therefore, the present results should be regarded as preliminary evidence for learning-related Hp–ST reorganization, and larger cohorts together with causal circuit manipulations will be needed to establish its functional role and generalizability.

## Figures and Tables

**Table 1:** FDR-corrected statistics for learning-related changes in gamma-band Hp→ST information flow.

Pigeon	Task Stage	Raw *p*-Value	FDR-Adjusted *q*-Value
P014	Stage 1	0.0001	0.0008
	Stage 2	0.0016	0.0021
P021	Stage 1	0.0011	0.0018
	Stage 2	0.0004	0.0011
P025	Stage 1	0.0010	0.0018
	Stage 2	0.0101	0.0115
P090	Stage 1	0.0357	0.0357
	Stage 2	0.0002	0.0008

**Table 2:** Temporal dynamics of theta- and beta-band Hp→ST directed information flow during learning.

Subject	Statistical Indicators	Stage 1 Theta Band (4−8 Hz)	Stage 1 Beta Band (12−30 Hz)	Stage 2 Theta Band (4−8 Hz)	Stage 2 Beta Band (12−30 Hz)
P014	*R* ^2^	0.0201	0.0193	0.0332	0.1606
	*p*-value	0.3828	0.3993	0.2103	0.0043
P021	*R* ^2^	0.3329	0.4094	0.1259	0.1737
	*p*-value	0.0020	0.0004	0.0463	0.0176
P025	*R* ^2^	0.0068	0.0251	0.0106	0.0136
	*p*-value	0.6114	0.3285	0.5878	0.5390
P090	*R* ^2^	0.1274	0.1075	0.0805	0.1684
	*p*-value	0.0238	0.0389	0.0536	0.0028

## Data Availability

The minimal dataset supporting the findings of this study has been provided with the submission. The full datasets are not publicly available because they form part of ongoing research and include data associated with studies that have not yet been published. Additional data are available from the corresponding author upon reasonable request.

## Abbreviations

The following abbreviations are used in this manuscript:

Hp	Hippocampus
ST	Striatum
LFP	Local field potential
RL	Reinforcement learning
MB	Model-based
MF	Model-free
RW-RL	Rescorla–Wagner reinforcement learning model
BIC	Bayesian information criterion
MAP	Maximum a posteriori estimation
PTE	Phase transfer entropy
VTE	Vicarious trial and error
